# Transcription factor Creb3l1 regulates the synthesis of prohormone convertase enzyme PC1/3 in endocrine cells

**DOI:** 10.1111/jne.12851

**Published:** 2020-04-21

**Authors:** Mingkwan Greenwood, Alex Paterson, Parveen Akhter Rahman, Benjamin Thomas Gillard, Sydney Langley, Yasumasa Iwasaki, David Murphy, Michael Paul Greenwood

**Affiliations:** ^1^ Translational Health Sciences Bristol Medical School University of Bristol Bristol UK; ^2^ Health Care Center Kochi University Kochi Japan

**Keywords:** dehydration, pituitary, POMC, prohormone processing, supraoptic nucleus, transcription

## Abstract

Transcription factor cAMP responsive element‐binding protein 3 like 1 (Creb3l1) is a non‐classical endoplasmic reticulum stress molecule that is emerging as an important component for cellular homeostasis, particularly within cell types with high peptide secretory capabilities. We have previously shown that Creb3l1 serves an important role in body fluid homeostasis through its transcriptional control of the gene coding for antidiuretic hormone arginine vasopressin in the neuropeptide‐rich magnocellular neurones of the supraoptic nucleus. In response to osmotic stimuli such as dehydration, vasopressin magnocellular neurones undergo remarkable transcriptome changes, including increased Creb3l1 expression, to ensure that the supply of vasopressin meets demand. To determine where else Creb3l1 fits into the secretory cell supply chain, we performed RNA‐sequencing of *Creb3l1* knockdown anterior pituitary mouse corticotroph cell line AtT20. The target chosen for further investigation was *Pcsk1*, which encodes proprotein convertase enzyme 1 (PC1/3). PC1/3 is crucial for processing of neuropeptides and peptide hormones such as pro‐opiomelanocortin (POMC), proinsulin, proglucagon, vasopressin and oxytocin. Viral manipulations in supraoptic nuclei by over‐expression of *Creb3l1* increased *Pcsk1*, whereas *Creb3l1* knockdown decreased *Pcsk1* expression. In vitro promoter activity and binding studies showed that Creb3l1 was a transcription factor of the *Pcsk1* gene binding directly to a G‐box motif in the promoter. In the dehydrated rat anterior pituitary, *Creb3l1* and *Pcsk1* expression decreased in parallel compared to control, supporting our findings from manipulations in AtT20 cells and the supraoptic nucleus. No relationship was observed between *Creb3l1* and *Pcsk1* expression in the neurointermediate lobe of the pituitary, indicating a different mechanism of PC1/3 synthesis by these POMC‐synthesising cells. Therefore, Creb3l1, by regulating the expression of *Pcsk1*, does not control the processing of POMC peptides in the intermediate lobe.

## 1 INTRODUCTION

cAMP responsive element‐binding protein 3 like 1 (Creb3l1), also known as OASIS, is a transcription factor in the CREB/ATF family. Because of the structural similarity to the endoplasmic reticulum (ER) stress inducer ATF6, early studies on Creb3l1 focused on its role in ER stress pathways.^1^ We now know that the actions of Creb3l1 are much more wide‐ranging than simply ER stress. Indeed, recent reports suggest that Creb3l1 is involved in cellular processes such as secretion, hormone synthesis, the formation of the extracellular matrix and cellular proliferation.^2^, ^3^, ^4^, ^5^ This is backed up by studies showing that Creb3l1 expression can be regulated by transforming growth factor beta, glucocorticoids and progesterone.^6^, ^7^, ^8^ These studies suggest that ER stress is one of many mechanisms through which Creb3l1 protein can be activated.

To act as a transcription factor, Creb3l1 is cleaved in the Golgi to liberate the transcriptionally active N‐terminal fragment, which then enters the nucleus to activate the transcription of target genes.^9^ Creb3l1 is expressed in a range of tissues, mostly secretory organs/cells, such as the pancreas, placenta, prostate gland, thyroid gland, gastrointestinal tract, osteocytes and neuroendocrine cells of the hypothalamus.^10^ We have previously reported increased Creb3l1 expression in the supraoptic nucleus (SON) of the rat and mouse is response to hyperosmotic stress,^11^ and identified Creb3l1 as a transcription factor for vasopressin (AVP) gene expression.^12^ The expression profile of Creb3l1, together with recent studies in endocrine/secretory tissue,^3^, ^13^ suggests that Creb3l1 may also regulate expression of genes involved in hormone secretion.

To identify transcriptional targets of Creb3l1, we performed high throughput transcriptomic RNA sequencing to catalogue genes altered in expression in AtT20 cells by stable *Creb3l1* knockdown. AtT20 is a well‐characterised secretory cell line, derived from mouse anterior pituitary corticotroph cells. These cells express high levels of the pro‐opiomelanocortin (POMC) hormone precursor, which is processed by proteolytic cleavage into several mature biologically active peptides that are subsequently secreted.^14^ To direct our candidate search towards secretory cells, we compared the AtT20 cell gene list with previously published transcriptomic data from the dehydrated rat and mouse SON.^11^ Creb3l1 is increased in the SON by dehydration.^8^, ^12^, ^15^, ^16^ Thus, we considered genes that increased in the SON in response to dehydration and decreased in AtT20 cells following stable knockdown of *Creb3l1*. We theorised that this cross‐analysis would help to identify transcriptional targets of Creb3l1 in the hormone synthesis and secretory pathway.

From our transcriptomic comparisons, the *Pcsk1* gene, which encodes the proprotein convertase enzyme 1 (PC1/3), was chosen for further investigation. PC1/3 is predominantly expressed in neural and endocrine tissues^17^, ^18^, ^19^, ^20^, ^21^ and is crucial for processing of neuropeptides and peptide hormones such as POMC, proinsulin, proglucagon, AVP and oxytocin.^22^ However, to date, the knowledge on transcriptional regulation of *Pcsk1* gene in these systems remains little understood.

## 2 MATERIALS AND METHODS

### 2.1 Animals

Male Sprague–Dawley rats weighing 200‐300 g were used in the present study. Rats were housed under a 14:10 hour light/dark cycle (lights on 5.00 am) at a constant temperature of 22°C and a relative humidity of 50%‐60%. Rats had free access to food and tap water for at least 1 week prior to experimentation. Animal experiments were performed between 9.00 am and 2.00 pm Experiments were performed under a Home Office UK license held under, and in strict accordance with, the provision of the UK Animals (Scientific Procedures) Act (1986); they had also been approved by the University of Bristol Animal Welfare and Ethical Review Board.

### 2.2 Hyperosmotic experiments

To induce acute hyperosmotic stress, a single intraperitoneal injection (i.p) of 1.5 mL 100 g^‐1^ body weight of 1.5 mol L^‐1^ NaCl solution was performed. Rats were randomly allocated into one of six groups: control (0), 10 minutes, 30 minutes, 1, 2 and 4 hours after administration of hypertonic saline. After injection, rats were placed back in their home cages, and water, but not food, was removed for the duration of the experiment. The control group had access to food and water ad lib. throughout the experimental period. For chronic hyperosmotic stimulation, drinking water was removed for 3 days (dehydration) or replaced with 2% (w/v) NaCl solution for 7 days (salt loading). For RNA and protein samples, rats were killed by striking of the cranium. Tissues were removed and immediately frozen using powdered dry ice and stored at −80°C until used. The pituitary gland was collected whole or separated into anterior and neurointermediate lobe (NIL) using sterile scalpel blades. For immunofluorescence staining, rats were anaesthetised using pentobarbital and perfused transcardially with phosphate‐buffered saline (PBS) followed by 4% (w/v) paraformaldehyde/PBS. Brains and pituitaries were removed and post fixed in 4% (w/v) paraformaldehyde/PBS overnight at 4°C, then cryoprotected in 30% (w/v) sucrose/PBS for approximately 72 hours before being frozen over liquid nitrogen and stored at −80°C.

### 2.3 Introduction of viral vectors into the SON

The *Creb3l1* and non‐targeting short hairpin RNAs (shRNAs) (see Supporting information, Table S1) were cloned into pGFP‐A‐shAAV (OriGene, Rockland, MD, USA). Adeno‐associated viral particles (AAV1/2) were produced using a helper free packaging system (Cell Biolabs, San Diego, CA, USA) to a titer of 6 × 10^12^ genome copies mL^‐1^ as described previously.^23^ The production of the constitutively active (CA) Creb3l1 and green fluorescent protein (GFP) lentiviral vectors has been described previously.^12^ For SON injections, rats were anaesthetised by i.p administration of a medetomidine and ketamine mix and placed in a stereotaxic frame in the flat skull position. A 2‐cm rostral‐caudal incision was made to expose the surface of the skull. Two 1‐mm holes were drilled at co‐ordinates 1.3 mm posterior to bregma and 1.8 mm lateral to midline. A 5‐µL pulled glass pipette was positioned −8.8 mm ventral to the surface of the brain and 1 µL of virus was delivered into nuclei over 10 minutes. The glass pipette was fixed in position for a further 5 minutes to minimise back tracking of the virus. Following injections, the incision was closed and atipamezole was administered intramuscularly. After surgery, animals were individually housed in standard laboratory cages for 2‐3 weeks.

### 2.4 Cell culture

Mouse pituitary cell line AtT‐20/D16v‐F2 (# 94050406; Sigma, St Louis, MO, USA) was maintained in a humidified incubator at 37°C with 5% CO_2_ in Dulbecco's modified Eagle's medium (# D6546; Sigma), supplemented with 10% heat‐inactivated fetal bovine serum, 100 μg mL^‐1^ penicillin/streptomycin and 2 mmol L^‐1^ l‐glutamine. *Creb3l1* knockdown cell lines were established using lentivirus expressing shRNAs as described previously.^8^ The sequences of all oligonucleotides and primers used in this study are provided in the Supporting information (Table S1).

### 2.5 RNA sequencing

Total RNA was extracted from AtT20 cells stably transduced with lentivirus expressing *Creb3l1*‐shRNA1 or non‐targeting control shRNA using Direct‐zol RNA extraction kit (Zymo Research, Irvine, CA, USA). RNA samples (n = 5 for each group) were sent to Source Bioscience (Nottingham, UK) for sequencing. Amplified cDNA libraries were prepared from isolated RNA samples and sequenced using the Illumina HiSeq 4000 Sequencer (Illumina Inc., San Diego, CA, USA). Briefly, total RNA samples (RNA integrity values 9.3‐9.8) were enriched by hybridisation to bead‐bound oligo‐d(T) probes using the TruSeq Stranded mRNA kit (Illumina Inc.) to obtain poly(A)‐selected samples and apply unique barcode adapters for sequencing. The libraries were assessed for their quality using a Qubit dsDNA High Sensitivity DNA Kit (Invitrogen, Carlsbad, CA, USA) and Agilent 2100 Bioanalyzer (Agilent High Sensitivity DNA Kit; Agilent Technologies, Santa Clara, CA, USA). All samples were normalised to 3 nmol L^‐1^. The libraries were pooled and clustered and sequenced on a HiSeq 4000 sequencing platform (Illumina Inc.). Paired‐end library reads of greater than 30‐40 million were generated for each individual library. The data were then processed using rta and casava (Illumina Inc.), thus providing sets of compressed FASTQ files per library. All raw reads were pre‐processed for quality assessment, adaptor removal, quality trimming and size selection using the fastqc toolkit^24^ to generate quality plots for all read libraries. We adopted a phred30 quality cut‐off (99.9% base call accuracy).

RNA sequencing alignment and data analysis were all performed in house using our high‐performance computer; “Hydra”. Our pipeline makes use of bash and python scripting to accept RNA sequencing post‐trimmed data as input, before ultimately producing output tables of differentially expressed transcripts. Paired‐end (2 × 75‐bp) raw input data is initially aligned with star ^25^ to the thirty‐eighth iteration of the *Mus musculus* reference genome (GRCm38.p6). featurecounts ^25^ is used to generate read counts, using the ENSEMBL Mus.musculus.GRCm38.97 annotation for reference.^26^ Our pipeline then uses deseq2 (version 1.22.2)^27^ from the r bioconductor package (https://www.bioconductor.org) to call differential gene expression. All *P* values were adjusted for multiple testing using the procedure of Benjamini and Hochberg. The data has been deposited in NCBI's Gene Expression Omnibus and is accessible through GEO Series accession number GSE147978 (https://www.ncbi.nlm.nih.gov/geo/query/acc.cgi?acc=GSE147978).

### 2.6 Luciferase assay

Luciferase assays in BE(2)‐M17 human neuroblastoma cells were performed as described previously.^28^ For experiments in AtT20 cells, the human PC1/3 promoter (−2047 to +205 bp) was cloned into pGL4 luciferase plasmid (Promega, Madison, WI, USA) with *Kpn*I and *Hind*III*.* Deletion constructs of the of *Pcsk1* promoter were generated by restriction digestion (*Hind*III with *Pst*1 for −1148 to +205 bp, *Bam*HI for −767 to +205 bp, *Bgl*II for −81 to +205 bp). For G‐box deletion, oligonucleotides containing −81 to +25 bp with and without G‐box, and −42 to +25 bp regions of *Pcsk1* promoter and their complementary strands were synthesised, then annealed together and cloned into pGL4 plasmid. To transfect DNA into cells lipofectamine LTX plus reagent (Invitrogen) was used. Accordingly, 400 000 cells were seeded into 12‐well plates. The total amount of plasmid used per well for co‐transfections was 1 µg (0.1 µg of promoter plasmid and 0.9 µg of Creb3l1‐expressing plasmid with 2 ng of pRL CMV‐Renilla luciferase control reporter vector; Promega). At 24 hours after transfection, luciferase assays were performed using Dual‐Luciferase^®^ Reporter Assay kit (Promega). Luciferase activity was measured in triplicate using a Lumat LB 9507 Luminometer (Berthold Technologies, Bad Wildbad, Germany). For chemical treatment, cells were transfected with 1 µg of plasmid containing −2047 to +205 bp of *Pcsk1* promoter and 2 ng of pRL CMV‐Renilla luciferase control reporter vector for 24 hours, then pretreated with 500 µmol L^‐1^ 3‐isobutyl‐1‐methylxanthine (IBMX; Sigma) for 15 minutes followed by 10 µmol L^‐1^ forskolin (FSK; Sigma) for 4 hours.

### 2.7 Quantitative polymerase chain reaction (PCR)

SON samples were collected and total RNA extracted as described previously.^12^ For pituitary samples, 200 µL of TRI reagent (Sigma) was added to frozen pituitaries and tissue was homogenised in 1.5‐mL Biomasher tubes (Takara, Kusatsu, Japan). For cells, 400 000 cells were seeded into 12‐well plates. At the time of collection, culture media was removed and 350 µL of TRI reagent was added to the well and incubated at room temperature for 5 minutes. The samples were centrifuged at 16 000 *g* for 1 minute to remove debris. RNA extractions were performed using a Direct‐zol RNA extraction kit (Zymo Research) and concentrations were determined by NanoDrop (Thermo Fisher Scientific, Waltham, MA, USA). For cDNA synthesis total RNA (200 ng for SON; 500 ng for cells and pituitary tissue) was reverse transcribed using the QuantiTect reverse transcription kit (Qiagen, Valencia, CA, USA). The cDNA from reverse transcription reactions was diluted 1:4 with water and used as a template for subsequent PCRs, which were carried out in duplicate using PowerUp SYBR Green master mix (# A25742; Thermo Fisher Scientific) on an StepOnePlus Real‐Time PCR system (Applied Biosystems, Foster City, CA, USA). For relative quantification of gene expression the 2^−ΔΔCT^ method was employed.^29^ The housekeeping genes used were *Rpl19* and *Sdha* (pituitary). Statistical analysis was performed on ΔCT values.

### 2.8 Western blotting

Cells were seeded into six‐well plates (800 000 cells well^‐1^). After 24 hours, cells were washed with PBS and harvested by scraping in 500 μL of radioimmunoprecipitation assay (RIPA) buffer supplemented with protease inhibitor cocktail (P8340; Sigma). Lysate was incubated on ice for 15 minutes with vortexing every 5 minutes. Debris was removed by centrifugation at 10 000 *g* for 10 minutes. For pituitary protein extraction, frozen pituitaries were homogenised in 100 µL of RIPA buffer in 1.5‐mL Biomasher tubes (Takara). Samples were incubated on ice for 30 minutes and centrifuged at 10 000 *g* for 10 minutes. Protein concentrations were determined by the Bradford assay.

Protein was subjected to sodium dodecyl sulphate‐polyacrylamide gel electrophoresis, then transferred to 0.45 µm polyvinylidene fluoride membrane (Millipore, Billerica, MA, USA). Membranes were blocked using 5% (w/v) skimmed milk in Tris‐buffered saline containing 0.05% (v/v) Tween 20 (TBS‐T) (except Creb3l1: 3% [w/v] bovine serum albumin [BSA]/TBS‐T) for 1 hour at room temperature. Primary antibodies were diluted in 2.5% (w/v) skimmed milk in TBS‐T (except for Creb3l1: 1% [w/v] BSA/TBS‐T) and incubated at 4˚C overnight. Secondary antibodies conjugated with horseradish‐peroxidase were diluted in 2.5% (w/v) skimmed milk in TBS‐T (except for Creb3l1: 1% [w/v] BSA/TBS‐T) and incubations were performed for 1 hour at room temperature. The signal was visualised by chemiluminescence using Supersignal West Dura chemiluminescent substrate (Thermo Fisher Scientific) using a genesys imaging system (Syngene, Cambridge, UK). Band intensity was determined using quantity one (Bio‐Rad, Hercules, CA, USA). The primary antibodies used were goat polyclonal anti‐N‐terminal Creb3l1 (dilution 1:500; AF4080; R&D Systems, Minneapolis, MN, USA), rabbit polyclonal anti‐prohormone convertase PC1/3/PC3 (dilution 1:1000: AB10553; Millipore), rabbit polyclonal anti‐POMC (dilution 1:5000; ab94446; Abcam, Cambridge, MA, USA), mouse anti‐Tubulin (dilution 1:10 000; MMS‐489P; Covance, Princeton, NJ, USA) and mouse polyclonal anti‐GAPDH (dilution 1:10 000; sc‐32233; Santa Cruz Biotechnology, Santa Cruz, CA, USA).

### 2.9 Chromatin immunoprecipitation assay

AtT20 cells (20 million cells) were grown in a 15‐cm plate for 24 hours. Cells were pre‐treated with 500 µmol L^‐1^ IBMX for 15 minutes before adding 10 µmol L^‐1^ FSK. Cells were collected after 20 hours and processed following the instruction of SimpleChIP^®^ Plus Enzymatic Chromatin IP Kit (# 9005; Cell Signaling Technology, Beverly, MA, USA). The antibody used for immunoprecipitation was rabbit anti‐Creb3l1 (2 µg; HPA024069; Sigma). Controls were performed with ChIP grade rabbit IgG (2 µg; ab171870; Abcam). The purified DNA was used for a quantitative PCR using mouse *Pcsk1* ChIP primers.

### 2.10 Immunofluorescence staining

Pituitaries were sliced in a cryostat at 40 µm thickness and placed onto cell culture inserts (Netwell; Costar Inc., Cambridge, MA, USA) in 12‐well plates containing PBS. Sections were washed three times for 5 minutes in PBS, then incubated in 10 mmol L^‐1^ sodium citrate buffer (pH6) at 95°C for 30 minutes. Tissue sections were placed at room temperature for 20 minutes for cooling and washed with PBS three times, 5 minutes each, then blocked and permeabilised in 3% (w/v) BSA prepared in 0.3% (v/v) triton‐X100/PBS (PBS‐T) for 30 minutes at room temperature. Primary antibodies were prepared in 1% (w/v) BSA/PBS‐T and incubated at 4°C for 48‐72 hours with constant rocking. After three 5‐minute washes in PBS, sections were incubated in darkness with secondary antibodies prepared in 1% (w/v) BSA/PBS‐T for 1 hour. Sections were washed twice with PBS and incubated with DAPI (2‐(4‐amidinophenyl)‐6‐indolecarbamidine dihydrochloride, 1 µg mL^‐1^) prepared in PBS for 1 minute. After washing with PBS, sections were mounted onto glass slides with 0.5% (w/v) gelatine (G9382; Sigma) and coverslipped with Vector‐Shields hard set mounting media (Vector Laboratories, Inc., Burlingame, CA, USA). Images were captured using an SP5‐II confocal laser scanning microscope attached to a Leica DMI 6000 inverted epifluorescence microscope (Leica Microsystems, Wetzlar, Germany). The primary antibodies were rabbit polyclonal anti‐POMC (dilution 1:5000; ab94446; Abcam) and goat polyclonal anti‐N‐terminal Creb3l1 (dilution 1:500; AF4080; R&D Systems). The secondary antibodies donkey anti‐goat‐Alexa 594 (dilution 1:500; Thermo Fisher Scientific) and donkey anti‐rabbit Alexa 488 (dilution 1:500; Thermo Fisher Scientific).

### 2.11 Statistical analysis

Replicates in all experiments are biological replicates. Statistical differences between two experimental groups were evaluated using independent‐sample unpaired Student's *t* tests. One‐way ANOVA with Tukey's or Dunnett's post‐hoc tests was used to determine the difference between more than two samples with only a single influencing factor. Two‐way ANOVA with a Bonferroni post‐hoc test was used to determine interactions between two independent variables on the dependent variable. Data are presented as the mean ± SEM *P* < 0.05 was considered statistically significant.

## 3 RESULTS

### 3.1 *Creb3l1* knockdown in AtT20 cells

**Figure 1:**
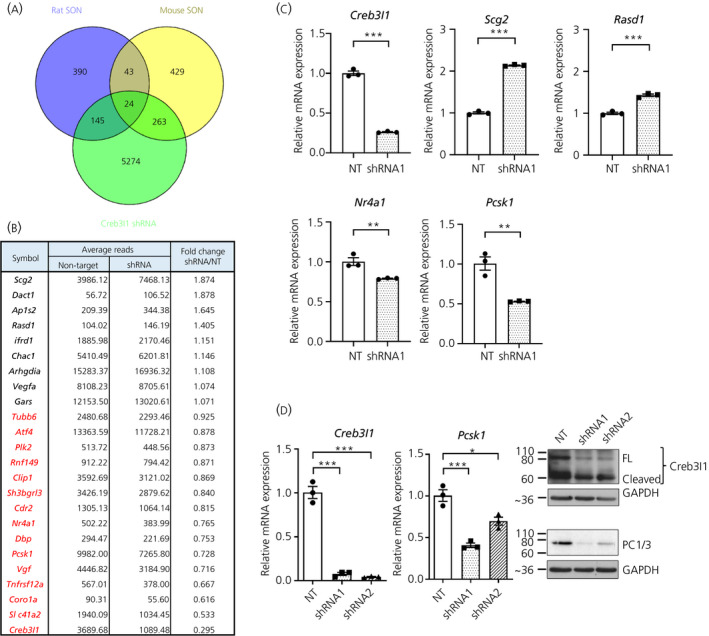
RNA sequencing of *Creb3l1* knockdown AtT20 cells. RNA sequencing was performed using RNA extracted from *Creb3l1* knockdown AtT20 cells induced by stable expression of *Creb3l1*‐shRNA1 (n = 5). A non‐targeting short hairpin RNA (shRNA) was used as a control (n = 5). The data were compared with previously published gene lists from microarrays of the rat and mouse supraoptic nucleus (SON) from control and dehydration (1.5‐fold cut off for the microarray data).^11^ A, Venn diagram showing the number of overlapping genes that changed in these data sets. B, List of genes that changes in all data sets. Genes in red change in the direction that implies Creb3l1 is a positive regulator (down in *Creb3l1*‐knockdown). C, Quantitative polymerase chain reaction (PCR) validation of RNA sequencing data for *Creb3l1*, *Scg2*, *Rasd1*, *Nr4a1* and *Pcsk1*. D, Quantitative PCR and western blotting analysis of *Creb3l1* and *Pcsk1* in *Creb3l1*‐knockdown AtT20 cells using two different *Creb3l1*‐shRNAs. NT, non‐targeting control; FL, full length; PC1/3, proprotein convertase enzyme 1. *P < 0.05; **P < 0.01; ***P < 0.001

To identify new transcriptional targets of Creb3l1, we performed RNA sequencing on our previously reported *Creb3l1* knockdown AtT20 stable cell line.^8^ The result showed that 5706 genes were significantly changed (*P*_adjusted_ < 0.01) in expression by *Creb3l1* knockdown (see Supporting information, Table S2). To select targets for further investigation, we compared these data with previously published microarray datasets from dehydrated mouse and rat SON where Creb3l1 increases in expression.^11^ Comparisons of mouse and rat microarray datasets revealed 67 genes that were changed (increased or decreased; >1.5 fold) in the SON of both rodents by dehydration (Figure 1A). Of these, 24 genes had differing expression in our RNA sequencing data from AtT20 cells (Figure 1B). Of these, 15 decreased in expression in *Creb3l1* knockdown AtT20 cells (Figure 1B, red) and 14 increased in the rodent SON following dehydration, suggesting that Creb3l1 could be a transcriptional regulator for these genes.

To validate these data from *Creb3l1* knockdown cells (*t* = 20.02, *P* < 0.0001), four genes were selected from the final list; secretogranin II (*Scg2*; *t* = 16.63, *P* < 0.0001), RAS dexamethasone‐induced 1 (*Rasd1*; *t* = 5.24, *P* = 0.0008), nuclear receptor subfamily 4, group A, member 1 (*Nr4a1*; *t* = 4.97, *P* = 0.008) and proprotein convertase subtilisin/kexin type 1 (*Pcsk1*; *t* = 7.514, *P* = 0.002), all of which successfully validated in AtT20 knockdown cells by quantitative PCR (Figure 1C). The gene picked for further investigation was *Pcsk1*, which encodes proprotein convertase enzyme PC1/3. To further confirm our data, a second *Creb3l1*‐shRNA cell line (shRNA2) was made in AtT20 cells (Figure 1D). Quantitative PCR analyses confirmed a decrease in both *Creb3l1* (*F*_2,6_ = 97.46, *P* < 0.0001) and *Pcsk1* (*F*_2,6_ = 45.08, *P* = 0.0002) mRNA expression in *Creb3l1* knockdown cells and western blotting at the protein level (Figure 1D). Our choice of PC1/3 was also informed by the literature as PC1/3 is important in prohormone cleavage of both POMC (AtT20 cells^30^) and AVP (SON^31^), suggesting a conserved functional role across these two experimental systems.

### 3.2 Effect of Creb3l1 on *Pcsk1* expression in the SON

**Figure 2:**
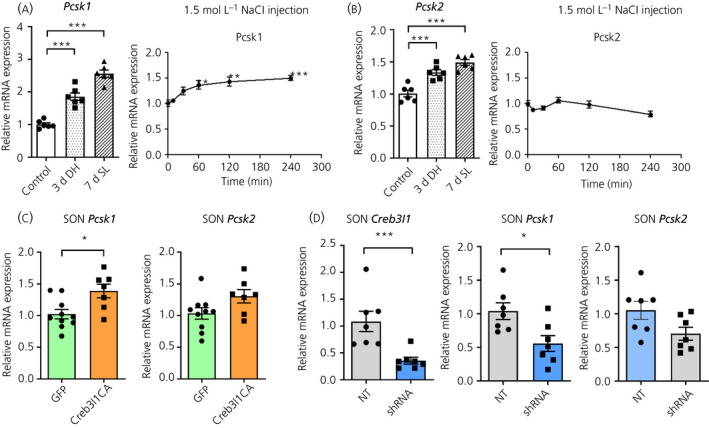
*Pcsk1* expression in the rat supraoptic nucleus (SON) during hyperosmotic stimulation and viral manipulations of *Creb3l1* expression. A, B, mRNA expression of (A) *Pcsk1* and (B) *Pcsk2* was investigated in the SON of control, 3‐day (3d) dehydrated and 7‐day (7d) salt loaded rats (n = 6) and rats i.p. injected with 1.5 mol L^‐1^ NaCl solution (1.5 mL 100 g^‐1^ body weight) (n = 5). C, Quantitative polymerase chain reaction (PCR) analysis of *Pcsk1* and *Pcsk2* for rat SON bilaterally injected with lentivirus expressing Creb3l1 CA. The successful expression of Creb3l1CA (n = 10 for GFP, n = 7 for Creb3l1 CA). D, Adeno‐associated virus expressing *Creb3l1* short hairpin RNA (shRNA) was unilaterally injected into rat SON. RNA was extracted from the SON 3 weeks after injection. The mRNA expression level of *Creb3l1*, *Pcsk1* and *Pcsk2* was investigated by quantitative PCR (n = 7). SON, supraoptic nucleus; DH, dehydration; SL, salt loaded; CA, constitutively active form; GFP, green fluorescent protein; NT, non‐targeting control. *P < 0.05; **P < 0.01; ***P < 0.001

We have previously reported increased *Creb3l1* expression in the rat SON following 3 days of dehydration, 7 days of salt loading and during an acute time course following hypertonic saline injection.^8^ Here, we show that *Pcsk1* abundance also increases (chronic *F*_2,15_ = 85.63, *P* < 0.0001; acute *F*_5,24_ = 9.18, *P* < 0.0001) in the SON in the same experimental models (Figure 2A). We also looked at the closely related proprotein convertase *Pcsk2* (Figure 2B), which is expressed in the SON.^18^ The abundance of *Pcsk2* mRNA increased (*F*_2,15_ = 27.27, *P* < 0.0001) in the SON of dehydrated and salt loaded rats compared to water replete controls. There was a significant effect (*F*_5,24_ = 3.62, *P* = 0.014) of NaCl injection on *Pcsk2* mRNA, although post‐hoc analysis found no time point‐specific differences, perhaps indicating different mechanisms regulating these PC family members in the SON.

To investigate the relationship between *Creb3l1* and *Pcsk1* expression in vivo, we used viral‐mediated gene transfer to either overexpress or to knockdown *Creb3l1* in the SON of control rats. Validation of *Creb3l1* overexpression by quantitative PCR has been confirmed alongside concomitantly increased AVP mRNA expression in these samples.^12^ Here, we show that overexpression of *Creb3l1* in the SON increases (*t* = 2.80, *P* = 0.014) *Pcsk1* and not *Pcsk2* mRNA expression (Figure 2C), at the same time as reducing (*t* = 4.97, *P* = 0.0003) endogenous *Creb3l1* expression decreases (*t* = 2.84, *P* = 0.015) *Pcsk1* but not *Pcsk2* mRNA expression in the SON (Figure 2D).

### 3.3 Creb3l1 positively regulates transcription of *Pcsk1* by binding to a G‐box on its promoter

**Figure 3:**
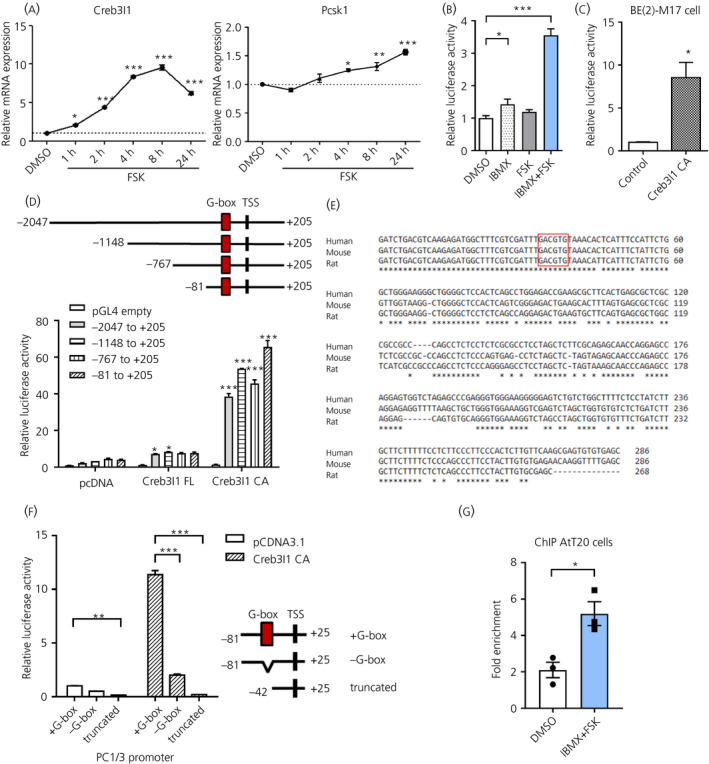
Creb3l1 binds onto the *Pcsk1* promoter to activate its transcription. A, Quantitative polymerase chain reaction (PCR) analysis of *Creb3l1* and Pcsk1 in AtT20 cells treated with 10 µmol L^‐1^ forskolin (FSK) at various time points. B, The effect of cAMP on *Pcsk1* promoter activity was determined via a luciferase assay. AtT20 cells were transfected with pGL4 containing −2047 to +205 bp *Pcsk1* promoter and subsequently treated with 10 µmol L^‐1^ FSK and/or 500 µmol L^‐1^ 3‐isobutyl‐1‐methylxanthine (IBMX). Samples were collected 4 hours after treatments. C, Effect of Creb3l1 CA on *Pcsk1* promoter activity in BE(2)‐M17 cells. D, Luciferase assay on AtT20 cells transfected with pGL4 plasmids containing various sizes of the *Pcsk1* promoter (as shown in the diagram) and plasmids expressing Creb3l1FL or Creb3l1CA. Empty pcDNA3.1 plasmid was used as a control. E, Sequence alignment of −81 to +205 bp *Pcsk1* promoter from human, mouse and rat. The red box indicates a potential Creb3l1‐binding site. F, *Pcsk1* promoter activity was determined by the luciferase assay in AtT20 cells transfected with pGL4 plasmids containing *Pcsk1* promoter with or without the G‐box together with the plasmid expressing Creb3l1 CA. G, ChIP assay using anti‐Creb3l1 antibody was performed in AtT20 cells treated with 10 µmol L^‐1^ FSK and 500 µmol L^‐1^ IBMX for 20 hours. Dimethyl sulphoxide (DMSO) was used as a vehicle control. n = 3 per group. TSS, transcription start site; FL, full‐length; CA, constitutively active form; NT, non‐targeting control. *P < 0.05; **P < 0.01; ***P < 0.001

The relationship between Creb3l1 and *Pcsk1* expression in different models suggested that Creb3l1 may directly regulate transcription of the *Pcsk1* gene. We have previously shown that *Creb3l1* mRNA and protein expression increases in response to increasing intracellular cAMP levels in AtT20 cells^8^ and, in the present study, we show increased *Creb3l1* expression (*F*_5,12_ = 353.2, *P* < 0.0001). Here we show that *Pcsk1* mRNA expression is increased (*F*_5,12_ = 24.26, *P* < 0.0001) at 4, 8 and 24 hours after treatment with FSK (Figure 3A). With the knowledge that endogenous *Pcsk1* is activated by cAMP pathways in these cells, we transfected AtT20 cells with a *Pcsk1* promoter luciferase reporter construct and treated the cells with IBMX and/or FSK and observed a significant effect of treatment (*F*_3,8_ = 74.53, *P* < 0.0001). FSK treatment alone did not increase luciferase activity by 4 hours, whereas treatment with the phosphodiesterase inhibitor IBMX, to quench the degradation of cAMP, increased luciferase activity (Figure 3B). FSK treatment potentiated IBMX induction of *Pcsk1* promoter activity. High levels of phosphodiesterase activity have previously been reported in AtT20 cells, consistent with these findings.^32^

Next, the effects of Creb3l1 on *Pcsk1* promoter were examined in two different cell lines. The luciferase assay on BE(2)‐M17 cells (Figure 3C) showed that Creb3l1 can activate (*t* = 4.17, *P* = 0.014) the *Pcsk1* promoter. To identify potential binding sites for Creb3l1 within the *Pcsk1* promoter, we navigated along the *Pcsk1* promoter using a series of enzyme directed deletions to truncate this promoter to within 81 bp of the transcriptional start site. Luciferase activity was measured following overexpression of either full‐length Creb3l1 (FL) or a CA mutant form of Creb3l1CA (Figure 3D). The result showed that both Creb3l1 FL and CA activated *Pcsk1* promoter activity (Promoter size *F*_4,30_ = 205.9, *P* < 0.0001, treatment *F*_2,30_ = 1708, *P* < 0.0001, interaction *F*_8,30_ = 129.3, *P* < 0.0001), supporting a role for Creb3l1 in *Pcsk1* transcription. Truncating the *Pcsk1* promoter had no effect on Creb3l1‐mediated luciferase activity, suggesting that a binding site might be located close to the transcriptional start site. Alignment of nucleotides −81 to +205 bp from human, mouse and rat *Pcsk1* genes showed that the proximal promoter was highly conserved (Figure 3E). The direct interactions of Creb3l1 with the rat AVP promoter are mediated by interactions with G‐box sequence (GCCCACGTGTGT).^12^ Here, we have identified a similar core G‐box motif GACGTG within the *Pcsk1* promoter. This sequence has recently been validated as a CrebA (Creb3l1‐orthologue)‐binding site in *Drosophila*.^13^ We subsequently made luciferase reporter constructs containing the *Pcsk1* promoter (+G‐box), a deletion mutant (−G‐box) and a further truncation of the promoter (truncated) (Figure 3F). Removal of the G‐box sequence prevented activation by Creb3l1 (promoter deletion *F*_2,12_ = 812.2, *P* < 0.0001, treatment *F*_1,12_ = 942.3, *P* < 0.0001, interaction *F*_2,12_ = 616.4, *P* < 0.0001) showing that this *cis*‐acting motif is essential for Creb3l1 activation of *Pcsk1*. Interestingly, decreased promoter activity was also observed in pcDNA control transfections, perhaps reflecting the basal activity of endogenous Creb3l1 in AtT20 cells.

To confirm direct binding of Creb3l1 on *Pcsk1* promoter, we performed ChIP assays in AtT20 cells treated with FSK and IBMX, with dimethyl sulphoxide (DMSO) treated cells acting as controls. A greater enrichment (*t* = 3.95, *P* = 0.017) of the *Pcsk1* promoter was observed in Creb3l1 pull‐downs from FSK + IBMX treated cells compared to DMSO controls (Figure 3G), suggesting direct binding of Creb3l1 to the *Pcsk1* promoter in these cells.

### 3.4 Knockdown of *Creb3l1* decreases POMC expression in AtT20 cells

**Figure 4:**
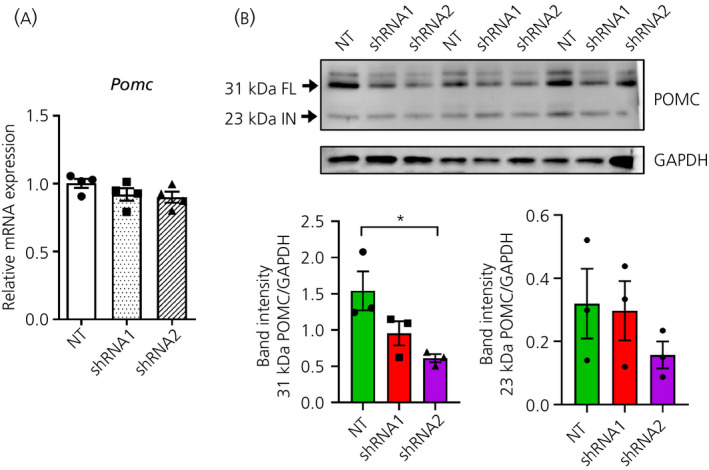
Effect of *Creb3l1* knockdown on pro‐opiomelanocortin (POMC) processing in AtT20 cells. A, Quantitative polymerase chain reaction analysis of *Pomc* mRNA expression in *Creb3l1* knockdown AtT20 cell lines compared to a non‐targeting control cell line. B, Western blot analysis of POMC in *Creb3l1* knockdown AtT20 cell lines compared to a non‐targeting control cell line. NT, non‐target control; FL, full length; IN, intermediate; shRNA, short hairpin RNA. *P < 0.05

To investigate downstream effects of a Creb3l1 regulated *Pcsk1* pathway, we first looked at POMC expression in our *Creb3l1* knockdown cell lines. AtT20 cells, being derived from anterior pituitary corticotroph cells, endogenously express and process POMC.^33^ There was no change in *Pomc* mRNA abundance following *Creb3l1* knockdown (Figure 4A), although western analysis showed that the 31‐kDa POMC prohormone was reduced by *Creb3l1* knockdown (*F*_2,6_ = 6.43, *P* = 0.03), whereas expression of the 23 kDa adrenocorticotrophic hormone (ACTH) biosynthetic intermediate did not change (Figure 4B).

### 3.5 Expression of Creb3l1, PC1/3 and POMC in the pituitary in response to dehydration

**Figure 5:**
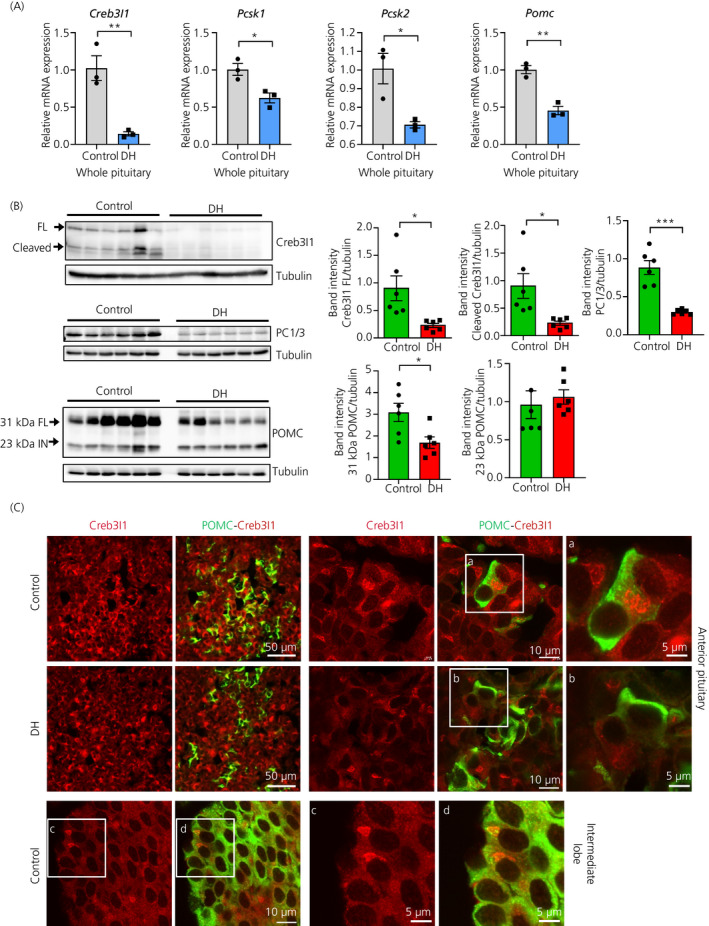
Creb3l1, proprotein convertase enzyme 1 (PC1/3) and pro‐opiomelanocortin (POMC) expression in the pituitary in response to dehydration. A, B, Expression of Creb3l1, PC1/3 and POMC was examined by (A) quantitative polymerase chain reaction and (B) western blotting. RNA and protein samples were extracted from whole pituitaries of control and 3‐day dehydrated rats. C, Immunofluorescence staining of Creb3l1 and POMC in pituitaries of control and 3‐day dehydrated rats. a, b, c and d are magnified images of the indicated boxes. DH, dehydration; FL, full‐length; IN, intermediate. *P < 0.05; **P < 0.01; ***P < 0.001

Stress‐induced plasma ACTH secretion is attenuated in dehydrated rats, which is not the result of altered *Pomc* mRNA expression suggesting altered processing of POMC.^34^ Therefore, we examined expression of Creb3l1, PCs and POMC in whole pituitaries from control and 3‐day dehydrated rats (Figure 5A). Quantitative PCR showed decreased mRNA expression for *Creb3l1* (*t* = 8.05, *P* = 0.001), *Pcsk1* (*t* = 3.52, *P* = 0.024), *Pcsk2* (*t* = 3.97, *P* = 0.017) and *Pomc* (*t* = 6.22, *P* = 0.003) in pituitaries from 3‐day dehydrated compared to control rats. Similarly, dehydration resulted in reduced PC1/3 (*t* = 6.35, *P* < 0.0001), POMC (*t* = 2.82, *P* = 0.018) and Creb3l1 (full‐length *t* = 6.92, *P* < 0.0001; cleaved *t* = 2.96, *P* = 0.014) steady‐state protein levels (Figure 5B). To confirm coexpression of Creb3l1 and POMC protein in pituitary corticotroph cells, immunofluorescence staining was performed on pituitaries from control and dehydrated rats (Figure 5C). The images show the expression of Creb3l1 in both POMC‐positive cells and POMC‐negative cells of the anterior pituitary in control and dehydrated rats. In the intermediate lobe of the pituitary, only a small proportion of POMC positive cells express Creb3l1.

### 3.6 Expression of Creb3l1, PC1/3 and POMC in neurointermediate and anterior lobes of the pituitary

**Figure 6:**
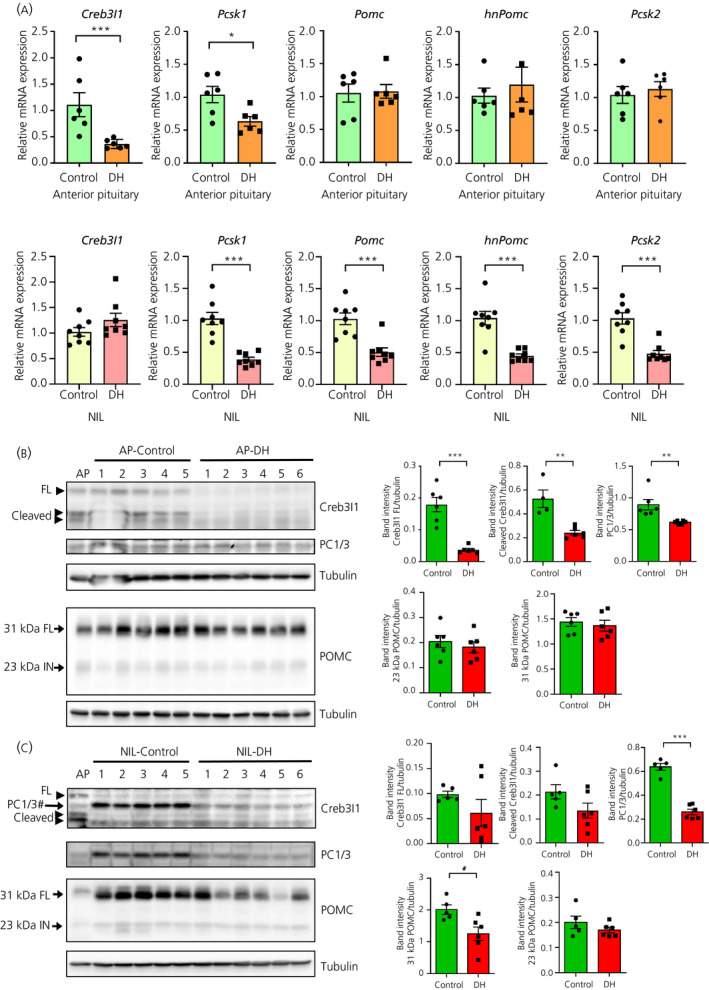
Creb3l1, proprotein convertase enzyme 1 (PC1/3) and pro‐opiomelanocortin (POMC) expression in the anterior pituitary (AP) and neurointermediate lobe (NIL). A, Quantitative polymerase chain reaction analysis of *Creb3l1*, *Pcsk1*, *Pomc*, *Pcsk2* and heteronuclear RNA of *Pomc* in anterior pituitary and NIL of control and 3‐day dehydrated rats. B, C, Western blot showing protein expression of Creb3l1, PC1/3 and POMC in (B) AP and (C) NIL of control and 3 d dehydrated rats. #PC1/3 bands that were probed prior to Creb3l1 antibody. DH, dehydration; FL, full length; IN, intermediate; *P < 0.05; **P < 0.01; ***P < 0.001

Because Creb3l1, PC1/3 and POMC are expressed by cells of the intermediate and anterior lobes of the pituitary, we investigated expression in separated NIL and anterior lobes of the pituitary from control and 3‐day dehydrated rats. In the anterior lobe of the pituitary, dehydration decreased *Creb3l1* (*t* = 4.63, *P* = 0.0009) and *Pcsk1* (*t* = 2.823, *P* = 0.018) mRNA expression, consistent with our findings from the whole pituitary (Figure 6A). However, anterior pituitary *Pomc* mRNA, heteronuclear RNA (hnPomc) and *Pcsk2* showed no significant change in response to dehydration (Figure 6A). For the NIL, there was no change in *Creb3l1* mRNA expression in response to dehydration, whereas *Pcsk1* (*t* = 7.51, *P* < 0.0001), *Pomc* (*t* = 5.06, *P* = 0.0002), *hnPomc* (*t* = 6.18, *P* < 0.0001) and *Pcsk2* (*t* = 5.89, *P* < 0.0001) significantly decreased (Figure 6A). Western analysis of anterior pituitary showed a robust decrease of Creb3l1 (full‐length *t* = 6.16, *P* = 0.0001; cleaved *t* = 4.59, *P* = 0.0018) and PC1/3 (*t* = 3.37, *P* = 0.007) in response to dehydration, whereas POMC did not change (Figure 6B). Western blots of NIL proteins extracts showed decreased PC1/3 (*t* = 10.29, *P* < 0.001) and 31‐kDa POMC (*t* = 2.83, *P* = 0.02), whereas Creb3l1 protein did not change (Figure 6C). These data show that these genes are differentially regulated in the anterior lobe and NIL of the pituitary in response to dehydration.

## 4 DISCUSSION

The present study reveals Creb3l1 to be a transcription factor for the *Pcsk1* gene in neuroendocrine cells of the hypothalamus and corticotroph cells of the pituitary gland. Because alterations of *Pcsk1* gene expression have been reported in relation to many diseases, such as diabetes mellitus, obesity, Alzheimer's disease, Huntington's disease and Prader–Willi syndrome,^35^, ^36^, ^37^, ^38^ it is important to understand the basic mechanisms responsible for its regulation.

We performed RNA sequencing of *Creb3l1* knockdown AtT20 cells to identify genes regulated by this transcription factor. With such a large number of gene changes, we needed a method to identify promising target genes for further investigation. We reasoned that because Creb3l1 is increased in the SON by dehydration, target genes would also be increased in this model, and so we made comparisons with transcriptome catalogues from the dehydrated rat and mouse SON. A number of common genes were identified and validated, including *Nr4a1*, which was previously identified by us as a transcription factor regulating *Creb3l1* expression,^16^ *Scg2*, a potential sorting receptor that targets proteins to secretory granules, and *Rasd1*, a small G protein that we recently identified in AVP neurones of the hypothalamus.^39^ These genes are highly expressed in neuroendocrine cells of the hypothalamus, including the SON and paraventricular nucleus (PVN), although they were not subjected to further investigation at this time. Rather, in the present study, we focused on the *Pcsk1* gene that encodes the hormone‐processing enzyme PC1/3.

We have previously reported up‐regulation of the *Creb3l1* gene in the hypothalamus following dehydration, salt loading and hyperosmotic stress^12^ and, in the present study, we demonstrate increased *Pcsk1* expression under these same conditions. During dehydration, AVP biosynthesis increases,^40^, ^41^ and an additional demand for this peptide necessitates changes in cell components necessary for processing and secretion.^42^ Because the AVP prohormone can be processed by PC1/3 in vitro^31^ and in vivo,^43^ and PC1/3 is highly expressed in AVP and oxytocin neurones of the hypothalamus,^18^ it is not surprising that we see an increase in PC1/3 expression under conditions that stimulate the AVP system. When *Creb3l1* expression was manipulated up or down in the hypothalamus by viral‐mediated gene transfer, we found that there was a parallel increase or decrease in *Pcsk1* expression. Therefore, Creb3l1 not only regulates AVP transcription, but also regulates processing via increased *Pcsk1* expression, making it a key molecular component of AVP biosynthesis.

There is a considerable literature that describes the role of PC1/3 in prohormone processing and its tissue distribution. However, the transcriptional regulation of the *Psck1* gene is less well understood. Creb3l1 and PC1/3 expression are both upregulated by increased cellular cAMP levels. A cAMP‐responsive element has been identified on the *Pcsk1* promoter and CREB and activating transcription factor 1 have been proposed to activate *Pcsk1* promoter activity.^44^, ^45^ Here, we identify Creb3l1 as a transcriptional regulator of the *Pcsk1* gene binding at a consensus Creb3l1‐binding site (G‐box) in AtT20 cells. The Creb3l1 binding site (G‐box) was first described by computational analysis of human transcription factor binding,^46^ and we showed that Creb3l1 binds to a similar G‐box sequence within the AVP promoter to regulate its transcription.^12^ The identification of this core consensus G‐box sequence for Creb3l1 may lead the way for finding further gene targets for Creb3l1 in secretory cells.

To translate our in vitro findings for corticotroph cells in vivo, we performed studies on control and dehydrated rat pituitary glands. In the anterior lobe of the pituitary, 15%‐20% of cells are corticotroph cells that synthesise POMC. PC1/3 is highly expressed in pituitary corticotroph cells, which we show in the present study to express Creb3l1, where it co‐ordinates POMC processing to produce ACTH,^17^, ^30^, ^47^ an integral component of the hypothalamic‐pituitary‐adrenal (HPA) axis stress response. In response to stress, synthesis of corticotrophin‐releasing hormone (CRH) increases in parvocellular neurones of the PVN, being released from axon terminals in the median eminence into the portal vasculature that supplies the anterior pituitary to stimulate the release of ACTH,^48^ and, subsequently, corticosterone release for the adrenal cortex.^49^ Chronic dehydration increases circulating levels of corticosterone, although it does not alter ACTH.^34^, ^50^, ^51^ Furthermore, dehydration decreases CRH expression in PVN parvocellular neurones,^34^, ^52^ suggesting a dampening of the central axis stimulating ACTH secretion, a response also observed in salt loaded rats.^53^ Studies investigating the stress response in relation to hydration status have shown that both dehydration and salt loading attenuate stress‐induced plasma ACTH secretion.^34^, ^52^, ^54^, ^55^ This occurs without alterations to pituitary *Pomc* mRNA expression.^34^ We know now that the attenuated ACTH secretion in dehydration may result from decreased PC1/3 expression.

In patients deficient in PC1/3, circulating levels of ACTH can be decreased, implying impaired POMC processing in the pituitary when levels of this convertase are depleted.^56^, ^57^ We also found *Pcsk2* expression in the anterior pituitary. In some cases, PC1/3 and PC2 have overlapping preferences for particular cleavage sites.^58^ For example, studies in rat pituitary GH3 cells, which only express PC2, have shown that exogenously expressed POMC can be completely processed to ACTH‐related peptides.^59^ However, an absence of PC1/3, as found in *Pcsk1* knockout mice, makes the level of ACTH undetectable in the pituitary.^60^, ^61^ Therefore, decreased anterior pituitary PC1/3 expression likely alters the availability of ACTH and, as a result, the stress response in dehydrated animals. We present correlative data of PC1/3 and Creb3l1 expression in pituitary corticotroph cells, and propose that Creb3l1 co‐ordinates the observed dehydration‐induced changes to anterior pituitary PC1/3 expression.

In the NIL, PC1/3 expression does not correlate with changes in Creb3l1 expression, suggesting different mechanisms of transcriptional control for *Pcsk1* in this tissue. This is consistent with our observations that not all POMC cells in the intermediate lobe express Creb3l1. However, our investigation did reveal dramatic reductions in *Pcsk1* and *Pcsk2* expression in the NIL. The *Pcsk1* and *Pcsk2* genes are not known to be expressed in the neural lobe of the pituitary,^17^ although they are found in the intermediate lobe. The intermediate lobe of the pituitary consists of melanotrophs expressing POMC, where the more abundant PC2,^17^, ^47^ rather than PC1/3, controls processing of POMC to produce α‐melanocyte‐stimulating hormone (MSH) and β‐endorphin. α‐MSH is formed by a series of cleavage steps involving both PC1/3 and PC2,^62^ both of which decrease in the intermediate lobe following dehydration. Peptidomic studies on pituitaries from *Pcsk1* and *Pcsk2* knockout mice show that loss of PC1/3 can be compensated for PC2.^58^ In PC1/3 knockout mice, α‐MSH production is unchanged consistent with PC2 compensation but can also be decreased consistent with impaired POMC processing in the pituitary. Thus, our data imply that α‐MSH formation should decrease during dehydration as a result of a decline in the synthesis of both PC enzymes and POMC itself. A decrease in *Pomc* expression has previously been described in the rat NIL following salt loading,^63^ supporting our findings of the present study with respect to dehydration. Any physiological significance of these modifications to prohormone processing machinery in the intermediate lobe in a state of dehydration remains to be determined.

In the SON, Creb3l1 increases *Pcsk1* expression to cope with the increased biosynthesis of AVP being released from the posterior pituitary. We report a complete contrast of events regarding the PC systems in the intermediate lobe and anterior portions of the pituitary where processing of prohormones would appear to be blunted in dehydration. Thus, to maintain hydromineral balance in chronic dehydration, the activity of the hypothalamo‐neurohypophyseal system increases whereas the HPA axis becomes less responsive to stress. We propose that changes to the HPA axis act to enhance the survival capabilities of dehydrated animals by reducing their response to stressors and promoting social behaviours. It has been proposed that such a mechanism may have evolved to suppress fear and anxiety in animals approaching a communal water source where predators may be encountered to enable drinking of fluids to restore body water content.^64^ Therefore, both these mechanism act to restore the physiological needs of the animal.

To summarise, we have identified Creb3l1 as a transcription factor of the *Pcsk1* gene in both the hypothalamus and corticotroph cells, providing new understanding about how *Pcsk1* gene expression is controlled. This information may be useful for future applications in diseases related to altered *Pcsk1* expression. It is important to note that *Pcsk1* expression does not appear to be regulated by Creb3l1 in pituitary melanotrophs. Therefore, this newly identified transcriptional pathway may not be a universal mechanism regulating *Pcsk1* expression, and as such, should be assessed on a cell‐type‐specific basis.

## Supporting information

Table S1

Table S2

## Data Availability

The data that support the findings of this study are available from the corresponding author upon reasonable request.
