# Supplementary material for: Transcription factor Creb3l1 regulates the synthesis of prohormone convertase enzyme PC1/3 in endocrine cells
Source: J Neuroendocrinol. 2020 Apr 21;32(4):e12851. doi: 10.1111/jne.12851 (PMC7359860; doi:10.1111/jne.12851)
Supplement: Supplementary file 1 — Table S1 [file JNE-32-e12851-s001.docx]

| **Primer name** | **Primer sequence - forward** | | **Primer sequence - reverse** |
| --- | --- | --- | --- |
| Mouse RPL19 | ACAAGCTCTTTCCTTTCGGTG | | GGGATCCAACCAGACCTTCT |
| Mouse/rat Creb3l1 | GAGACCTGGCCAGAGGATAC | | GTCAGTGAGCAAGAGAACGC |
| Mouse Pcsk1 | TGTGTCTCTGATCTTGCTTCTTTTC | | AGTCCAACCTCTTTGCTCCAT |
| Mouse POMC | TACCCCAACGTTGCTGAGAA | | GACCTGCTCCAAGCCTAATG |
| Mouse Scg2 | GATGAAACGTTCAGGGCAGT | | GATGAAACGTTCAGGGCAGT |
| Mouse Rasd1 | CCCTCAGCGTTGTGCCTACT | | AAAGAGCGCACGGAACATCT |
| Mouse Nr4a1 | GCACAGCTTGGGTGTTGATGT | | GAGCCCGTGTCGATCAGTGAT |
| Rat RPL19 | GCGTCTGCAGCCATGAGTA | | TGGCATTGGCGATTTCGTTG |
| Rat Pcsk1 | AGTGTACTGCTTTCACCCTCT | | ACAAACTGCCTCTTCGCTTT |
| Rat Pcsk2 | GGAGGAAGAGGAATCCCGAG | | GCTGCAGATGTCCCAGAATG |
| Rat POMC | CGACAGAGCCTCAGCCA | | ATGGAGGTCTGAAGCAGGAG |
| Rat Sdha | GGCGGGATTCCCACTAACTA | | ACAGACCAGGCACAATCTGA |
| Mouse Pcsk1_ChIP | CGGGAATACCATCCAGTTCT | | CAGCCTTACCAACCAGAAT |
| **shRNA sequences (AtT20 cell lines)** | | | |
| Creb3l1-shRNA1 | | GAGTCGGATTTCCTCAACAAT | |
| Creb3l1-shRNA2 | | CGGCTCAATGACTGTGAAAGA | |
| Non-targeting shRNA | | ATCATGTTAGGCGTACGGACT | |
| **shRNA sequences (Rat – *in vivo*)** | | | |
| Creb3l1-shRNA | | AGACCTGAATGAGTCGGATTT | |
| Non-targeting shRNA | | AATTCTCCGAACGTGTCACGT | |
